# A Synthetic Dataset for Semantic Segmentation of Waterbodies in Out-of-Distribution Situations

**DOI:** 10.1038/s41597-024-03929-2

**Published:** 2024-10-10

**Authors:** Eleftherios Ioannou, Sainath Thalatam, Serban Georgescu

**Affiliations:** 1 Fujitsu Research of Europe, Slough, United Kingdom; 2 University of Sheffield, Sheffield, United Kingdom

**Keywords:** Water resources, Environmental impact

## Abstract

In the past decade, substantial global efforts have been devoted to the development of reliable and efficient solutions for early flood warning and monitoring. One of the most common strategies for tackling this challenge involves the application of computer vision techniques to images obtained from the numerous surveillance cameras present in urban settings today. While there are various datasets available for training and testing these techniques, none of them specifically addresses the issue of out-of-distribution (OoD) behavior. This issue becomes particularly critical when evaluating the reliability of these methods under challenging environmental conditions. Our work stands as the first attempt to bridge this gap by introducing a new, highly controlled synthetic dataset that encompasses the essential attributes required for analyzing OoD behavior. The very high correlation between the accuracy of artificial intelligence (AI) models trained on our synthetic dataset and models trained on real-world data proves our dataset’s ability to predict real-world OoD behavior reliably.

## Background & Summary

In light of growing environmental concerns in recent years and the pressing need to avert environmental disasters, the field of vision-based semantic segmentation for waterbodies has gained increasing importance. This task aims to identify water in images and plays a pivotal role in various applications such as flood mitigation, aquatic ecosystem monitoring, urban planning, and resource management. However, despite the progress in deep learning and scene understanding, semantic segmentation models face notable challenges in accurately identifying and segmenting water due to its inherent properties like reflectivity, roughness, color, waviness and depth, compounded by environmental factors like lighting and fogginess.

Except for the well-known and widely used large-scale annotated datasets^1–5^ that allow for the design and development of computer vision applications, it is only recently that efforts have been made to collect or capture images that include water or flood-related scenes. Sazara *et al*.^6^ attempt to both classify areas as flooded or non-flooded using an image classifier and segment flooded areas using superpixel-based methods and Fully Convolutional Neural Networks. As part of their work, they generate a small dataset of 253 manually annotated flood images. Similarly, Sarp *et al*.^7^ propose a system for detecting and segmenting floodwater if present in an image and release a dataset of 441 annotated roadway flood images. The more recent work of Pally *et al*.^8^ leverages data from multiple sources such as social media platforms, the Department of Transportation (DOT), the US Geological Survey (USGS), and online search engines to build a dataset of more than 9000 images with annotations of multiple object categories. The *ATLANTIS* dataset^9^ contains 5,195 pixel-wise annotated images including 56 labels of different waterbodies and water-related objects. This has been the most comprehensive attempt to create a suitable dataset for the task of semantic segmentation of waterbodies. Table 1 provides an overview of the datasets that contain water or flood-related images, that make segmentation of waterbodies and flood-related research plausible.

**Table 1:** Datasets comprising of images that include water or flooding.

Dataset	No. Images	No. Classes	Main Task
Pascal Context	19740 (899)	520	Semantic Segmentation
ADE20K	25000 (885)	3169	Scene Understanding
Mapillary Vistas dataset	25000 (∼600)	66	Semantic Segmentation
Gebrehiwot *et al*.	100	2	Flooding
Sazara *et al*.	253	2	Floodwater Segmentation
Sarp *et al*.	441	2	Floodwater Segmentation
ATLANTIS	5195	56	Segmentation of Waterbodies
Pally and Samadi	>9000	8	Object detection, Water Level, Water Region

For the largest datasets that contain many classes, the number of images in parenthesis represents the number of images that contain the label ‘water’.

Existing datasets are helpful for research but fall short in addressing out-of-distribution (OoD) cases crucial for safety-related applications such as flood early warning systems. Most available datasets focus on ‘normal’ conditions, thereby limiting the assessment of model performance in critical scenarios like severe weather conditions. No dataset contains annotations explicitly designed to capture the particular attributes of the water (e.g., reflectivity, color, level) or the surrounding environment (e.g., light intensity, fog density). Such information could provide immense insights into identifying the failure cases of the computer vision models and allow for their improvement.

**Fig. 1:**
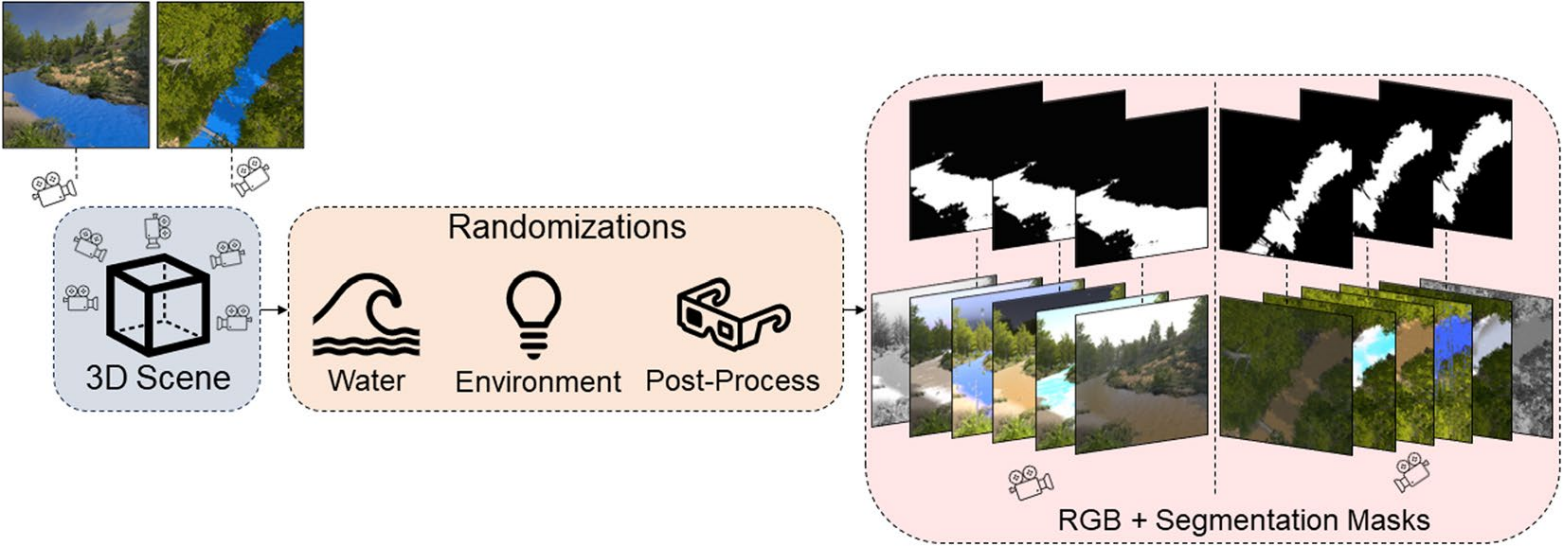
Overview of our synthetic dataset generator framework. A 3D scene includes multiple cameras facing waterbodies in the scene. Through a series of randomized parameters that control the appearance of the water, the environment and the applied post-process effects, RGB frames and their accompanied binary segmentation masks are generated.

In this paper, we present for the first time a large-scale highly controlled binary-labeled dataset that can be used to better understand and hopefully improve the OoD behaviour of segmentation models for water bodies. We named this dataset *FlOoD*^10^. To create *FlOoD*, we first define a list of relevant attributes that relate to the water and its surroundings and which potentially affect and degrade the performance of the current computer vision methods. We parameterize these attributes and implement a synthetic data generation pipeline that samples them in order to create a diverse dataset of labelled images (Fig. 1).

To gauge the effectiveness of our developed datasets, we examine the performance of state-of-the-art models that were originally designed, developed, and trained on standard semantic segmentation benchmark datasets, such as MS COCO^11^ and VOC^2^. The list of networks we investigate on real-world and computer-generated datasets includes DeepLabV3^12^, PSPNet^13^, CCNet^14^, OCRNet^15^, OCNet^16^, DANet^17^, ANNet^18^, EMANet^19^, GCNet^20^ and DNLNet^12^. We show that the accuracy of these models trained and tested on *FlOoD* correlates very well to that of models trained and tested on real datasets such as *ATLANTIS*. This implies that results obtained on *FlOoD* are good predictor of real-world OoD performance.

## Methods

### Dataset’s attributes

We design *FlOoD*^10^ with the goal of capturing relevant characteristics related to the water’s appearance, the surrounding environment’s manifestation in the scene, and the potential camera effects that might occur when monitoring waterbodies in real-world locations. Our attempt to identify and reproduce the attributes encountered in real-world imagery is inspired by the diverse recently-released dataset of *ATLANTIS* and the *Farson Digital Watercams* archive (https://www.farsondigitalwatercams.com/) that broadcasts live feeds from UK’s and Republic of Ireland’s waterways. All the defined attributes are summarized in Table 2. A short description and the value range are provided for every attribute. The assigned value range aims to serve both the implementation of the annotations that are generated for each rendered image and the implementation of the randomization activities that are performed during the generation process. For most of the attributes, we define the values to be in the range of 0.0 to 1.0; for example, a value of $0$ for water reflectivity corresponds to the water material being dull with no reflectivity of the surroundings, whereas a value of $1.0$ means that the surroundings are clearly reflected on the water’s surface. For the color of the water, a set of RGB values is pre-defined. For the water level, we pre-define a water level position that is considered normal (not very low and not flooded) and set the shift range to be from $-2$ to $+2$ meters. In addition, our pipeline supports the enablement of several post-process effects. These aim to resemble malfunctions of the live-feed cameras e.g., ISO Noise, Black & White, or different external conditions that might influence the quality of the imagery such as raindrops on the camera lens or bloom (bright highlights or dirty lens).

**Table 2:** The defined attributes for water, environment and post-processing effects used to generate *FlOoD*.

Attribute	Description	Values
**Water Attributes**		
Water Reflectivity	Degree of reflectiveness of surroundings on water’s surface	[0, 1]
Water Distortion	Degree of distortion of the reflections	[0, 1]
Water Level	Level of water relatively to a pre-defined “regular” position	[−2, 2]
Water Color	Color of the water (RGBA)	[0–255] × 4
Water Vertical Depth	Visibility of the underground underneath	[0,1]
Water Horizontal Depth	Visibility of the underground underneath in distance from the camera	[0,1]
Water Foaming	Degree of foaming on water’s surface	[0,1]
**Environment Attributes**		
Light Intensity	Intensity of the light in the scene	[0,1]
Fog Density	Amount of fog present in the scene	[0,1]
**Post-processing effects**		
ISO Noise	Simulates ISO noise/grain appearing in cameras	[True, False]
Black and White	Makes the rendered image black and white	[True, False]
Depth of Field	Simulates the focus properties of a camera lens	[True, False]
Bloom	Very bright highlights and dirt on lens effect	[True, False]
Raindrop on Lens	Simulates raindrops on camera’s lens effect	[True, False]

Although not included in the list of attributes, our generation process also supports the randomization of some other environmental factors–skybox, sky color, and rain/snow–to introduce more diversity. For the skybox, we pre-define a set of Skybox materials. To allow for shifts in sky color, we utilize a 3D skybox model to resemble clouds. For rain and snow, we implement particle systems and vary the intensity levels.

To ensure that the generated dataset includes images accurately representing extreme weather conditions, we manually adjusted the minimum and maximum values for each attribute. This tuning was performed to correspond to frames where the effect (e.g., light intensity) is at its maximum (or minimum) while still allowing the waterbody to be noticeable to the human eye. Each scene was carefully calibrated before the generation process. Manual inspection of the dataset, along with statistics on the coverage of attribute values, demonstrates that the complexity of real-world environments is adequately simulated.

### The environment

**Fig. 2:**
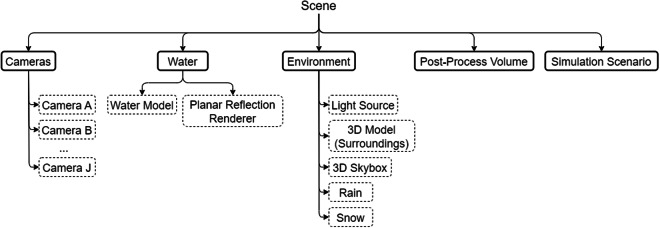
The Scene Hierarchy.

Multiple 3D scenes are created to capture a wide variety of scenarios and to accommodate diversity in the generated dataset. The composition of each scene is kept consistent and it is illustrated in Fig. 2. To capture a waterbody from multiple views, we place several cameras at different locations with a variety of viewing angles and rotations. We also place a Directional Light for the simulation of different lighting conditions. We utilize the Water System from *Staggart Creations* (https://assetstore.unity.com/packages/vfx/shaders/stylized-water-2-170386) that is accompanied by a Planar Reflection Renderer that aids the appearance of reflections on the water’s surface. The water model is placed in the scene according to the 3D model of the surrounding area (e.g., terrain, city, bridge). The Post-Process Volume contains all the post-process effects, while the Simulation Scenario is responsible for the control of the randomized simulations executed for each camera in the scene, as well as the regulation of a number of different parameters e.g., the number of frames that are captured.

It is important to note, that although we employed high-quality 3D assets that are designed to closely mimic real-world water bodies, including detailed textures and realistic water surface dynamics, a domain gap between synthetic and real images is inevitable. We would like to emphasize that, as proven by our experiments, this gap does not impact FlOoD’s performance as a proxy for real-world data.

### The randomizations

Our proposed synthetic dataset generator framework is illustrated in Fig. 1. In addition to providing annotations for each rendered frame and segmentation mask, we use the defined attributes and Unity Perception’s capacity to execute randomized simulations, to create randomizations for the generation of a large and diverse dataset. Through the Unity Perception package, it is possible to define an array of randomization activities that are performed during the lifecycle of a simulation. A simulation can last for a number of frames during which several parameters are varied. We make use of the attribute definitions we defined (Table 2) to create randomizers that sample from the given value range as a uniform or normal distribution, resulting in alterations of the scene and generation of various water, environment, and post-process effects. Figure 4a,b provide examples of frames generated with varying reflection intensity and varying water level respectively.

**Fig. 3:**
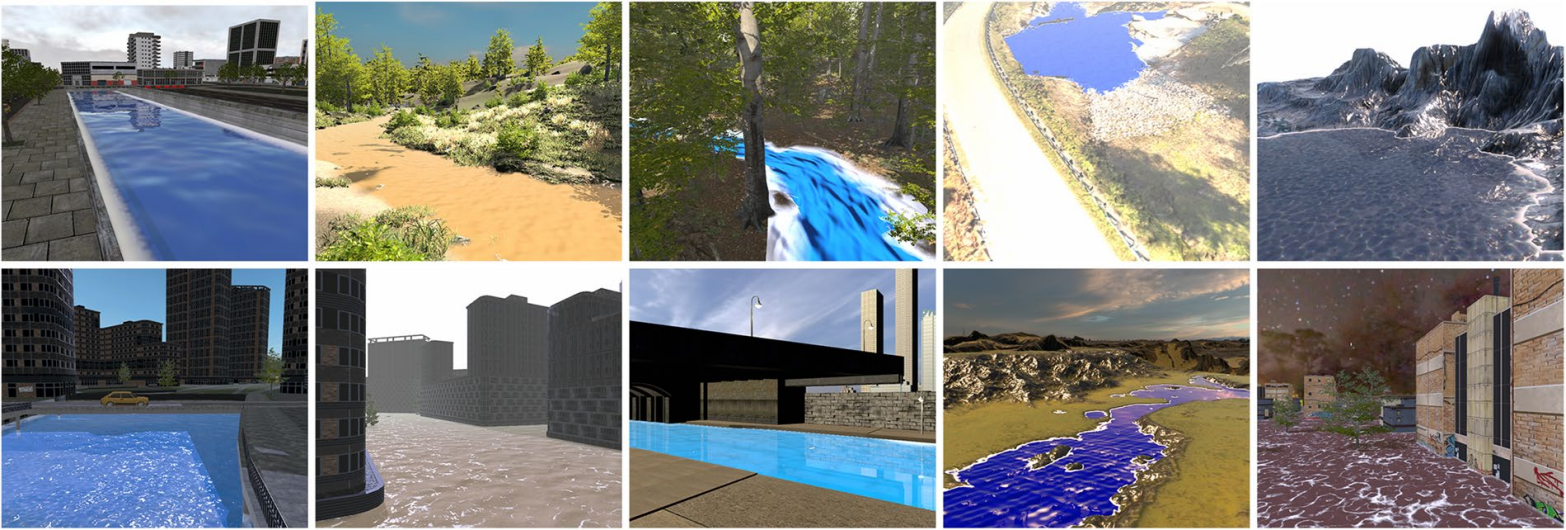
Sample frames from different scenes in *FlOoD*.

**Fig. 4:**
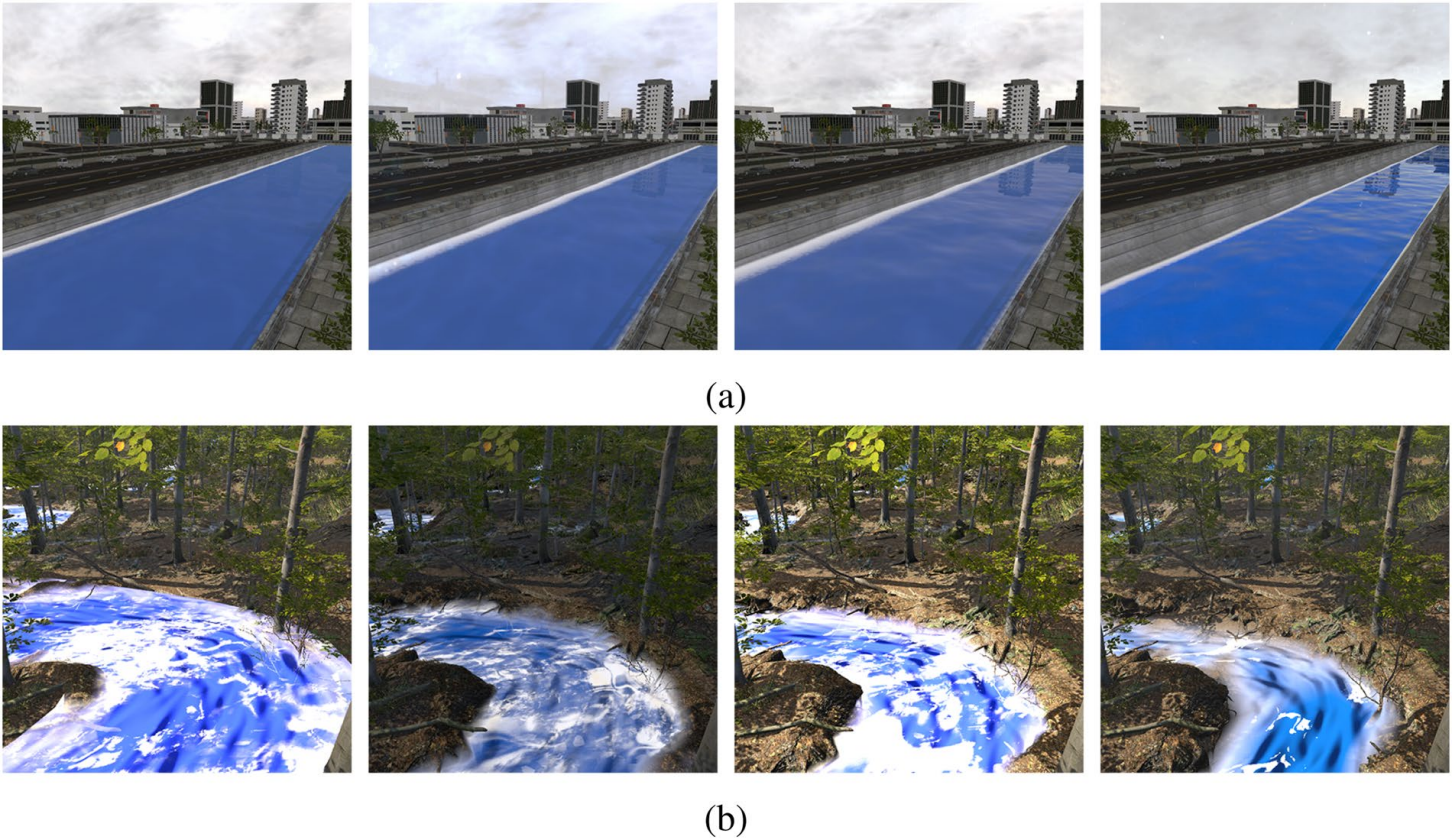
(**a**) Varying reflection intensity of the water. (**b**) Varying water level.

## Data Records

Data is available at figshare^10^. Sample frames from *FlOoD* are shown in Fig. 3.

The *FlOoD* comprises a collection of synthetic scenes, each carefully generated to encapsulate a unique combination of water and environmental attributes. The dataset contains frames from 11 different scenes, with each scene annotated with ground truth binary masks for water body segmentation. The Unity Perception package provides the capability to assign labels to 3D objects within a scene. In our dataset, all water objects are assigned one label, while all other objects receive a different label. Due to the nature of Unity’s 3D rendering pipeline, these binary labels are accurately generated. To validate the segmentation labels and verify their consistency, random frames from all the scenes were manually inspected. Our approach consistently generates images and binary segmentation masks that are pixel-level accurate.

Our large-scale dataset consists of 9200 frames; a training-test split is performed that results in the training set having 8100 images and the test set having 1100 images. The images are generated to be 640 $\times$ 640, facilitating their direct utilization for training purposes. The values for all the attributes related to water, environment and post-process effects (Table 2) are also provided for each generated frame. The values are in the range depicted in Table 2.

### FlOoD dataset

We split the dataset into train and test folders. The folder structure of each of the (train, test) directories is:where $s$ corresponds to the scene number, $c$ is the camera identification number, $i$ is the image’s name and $N$ is the number of images; $m$ represents the binary segmentation mask corresponding to each $i$, and the file *metadata.json* contains the attribute values for each of the frame. These correspond to the attributes defined in Table 2, where for the post-process effect key (“post_effect”), the string “none” denotes that no post-processing effect is applied, and the strings “iso_noise”, “black_and_white”, “depth_of_field”, “bloom”, and “raindrops_on_lens” denote the effect as described in Table 2.

## Technical Validation

This section outlines the experiments undertaken to demonstrate the correlation between real and synthetic datasets through a high Pearson’s correlation coefficient of segmentation. Moreover, we explore how different splits of the synthetic dataset, namely 2.65k and 4k images, affect the various model’s ability to perform water segmentation. For testing the performance of the models, we use both water insection over union (IoU) and pixel accuracy as main metrics.

### Dataset preparation

The real-world *ATLANTIS* dataset employed as reference in this research is constituted of 5,195 RGB images, which contain a substantial proportion of water bodies in 56 labels of different waterbodies and water-related bodies. We convert it to a binary mask (water and non-water). Both *FlOoD* and *ATLANTIS* were subjected to pre-processing to standardize the image size, normalize pixel intensities, and apply data augmentation techniques to increase the model robustness.

### Model training

To understand the correlation between the behaviour of models trained on real datasets such as *ATLANTIS* with the behaviour on *FlOoD*, we employed a wide range of state-of-the-art models: DeepLabV3, PSP-Net, CCNet, OCRNet, OCNet, DANet, ANNet, EMANet and GCNet. The results of five such experiments are reported in Table 3 where:

- Columns 1-2 show the test accuracy when training and testing on real data only
- Columns 3-4 show the test accuracy when training and testing on *FlOoD* only
- Columns 5-6, 7-8 and 9-10 show the test accuracy when testing on real world data but training on a combination of real and a varying amount of synthetic data

**Table 3:** Experimental results of training and testing various State-of-the-Art models on different combinations of real and synthetic data.

Dataset [Training-Test]										
Model	ATLANTIS -ATLANTIS		Synthetic -Synthetic		ATLANTIS+SyntheticFull ATLANTIS		ATLANTIS+Synthetic4k ATLANTIS		ATLANTIS+Synthetic2.65k ATLANTIS	
	Water IoU	Pixel Acc	Water IoU	Pixel Acc	Water IoU	Pixel Acc	Water IoU	Pixel Acc	Water IoU	Pixel Acc
DeepLabV3	0.853	0.948	0.836	0.932	0.847	0.946	0.845	0.945	0.853	0.948
PSPNet	0.825	0.937	0.819	0.922	0.818	0.932	0.824	0.934	0.833	0.938
CCNet	0.834	0.94	0.836	0.934	0.836	0.938	0.833	0.937	0.844	0.945
OCRNet	0.837	0.942	0.845	0.937	0.839	0.944	0.807	0.93	0.813	0.933
OCNet	0.826	0.935	0.833	0.934	0.828	0.936	0.826	0.935	0.829	0.936
DANet	0.73	0.883	0.783	0.901	0.819	0.928	0.811	0.927	0.817	0.93
ANNet	0.833	0.939	0.846	0.939	0.847	0.944	0.841	0.941	0.842	0.942
EMANet	0.847	0.946	0.855	0.94	0.846	0.944	0.816	0.932	0.817	0.93
GCNet	0.832	0.938	0.821	0.924	0.833	0.939	0.822	0.933	0.846	0.944
Average	0.824	0.934	0.830	0.929	0.835	0.939	0.825	0.935	0.833	0.938

The default training-validation-test split is used for the ATLANTIS dataset.

### Correlation analysis

To investigate the correlation between our synthetic dataset *FlOoD* and real dataset *ATLANTIS* we computed the Pearson’s correlation coefficient between the IoU scores obtained when training and testing all models on real data (Table 3 columns 1-2) and those when training and testing the same models on *FlOoD* (Table 3 columns 3-4).

**Fig. 5:**
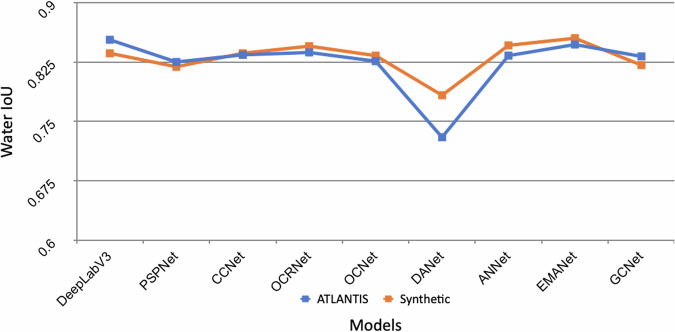
Strong correlation in the IoU metric between models trained on *ATLANTIS* and *FlOoD* datasets. This high correlation suggests that the synthetic data captures essential characteristics of real data, thereby validating its utility for training and evaluating models.

Our analysis, shown in Fig. 5, reveals a high Pearson’s correlation coefficient of 0.89, demonstrating a strong positive correlation between synthetic and real dataset performance. This high correlation implies that results obtained from OoD experiments performed using *FlOoD* are a very good proxy for the OoD behaviour of models trained on real-world datasets like *ATLANTIS* where such experiments are impossible to perform.

We further confirm the high correlation between our synthetic data and real data by a second experiment where we compare the accuracy on the ATLANTIS dataset of a model with randomly initialized weights with that of a model pre-trained on our synthetic dataset. As shown in Table 4, the accuracy of the latter far exceeds the accuracy of the former which provides additional evidence for the correlation between our dataset and real data.

**Table 4:** Experimental results of training state-of-the-art models with randomly initialized weights and training on *FloOD*, and testing on the ATLANTIS dataset.

Model	Dataset [Training-Test]			
	Random-ATLANTIS		Synthetic-ATLANTIS	
	Water IoU	Pixel Acc	Water IoU	Pixel Acc
DeepLabV3	0.406	0.617	0.642	0.824
PSPNet	0.444	0.768	0.613	0.822
CCNet	0.394	0.617	0.615	0.825
OCRNet	0.394	0.62	0.604	0.799
OCNet	0.244	0.379	0.58	0.814
DANet	0.228	0.362	0.623	0.809
ANNet	0.447	0.77	0.617	0.819
EMANet	0.432	0.727	0.604	0.805
GCNet	0.416	0.702	0.605	0.788
Average	0.3783	0.618	0.6114	0.8117

## Usage Notes

We offer a rich synthetic dataset for semantic segmentation of waterbodies in OoD scenarios. All the attributes defined in Table 2 can be found in the metadata files. The dataset comes split into a train set and test set which have been used to obtain the results show in Table 3. However, we expect users to define new splits based on various combinations of the provided attributes.

While the main aim of *FlOoD* is to allow researchers to assess the OoD performance of their models, it is also possible to use *FlOoD* for data augmentation purposes. The averages shown at the bottom of Table 3 for columns 5-6, 7-8 and 9-10 indicate that a small performance increase of up to 0.5 percentage point can be indeed achieved in this way.

## Data Availability

The code that was used to generate the data uses commercial assets and functions of the commercial Unity package. For reference, the code is available at https://github.com/FujitsuResearch/SyntheticWaterBodies.
